# Transcriptomic Effects of Acute Ultraviolet Radiation Exposure on Two *Syntrichia* Mosses

**DOI:** 10.3389/fpls.2021.752913

**Published:** 2021-10-28

**Authors:** Jenna T. B. Ekwealor, Brent D. Mishler

**Affiliations:** ^1^Department of Integrative Biology, University of California, Berkeley, Berkeley, CA, United States; ^2^The University and Jepson Herbaria, University of California, Berkeley, Berkeley, CA, United States

**Keywords:** *Syntrichia caninervis*, *Syntrichia ruralis*, bryophyte, desiccation tolerance, transcriptomics, UV radiation tolerance

## Abstract

Ultraviolet radiation (UVR) is a major environmental stressor for terrestrial plants. Here we investigated genetic responses to acute broadband UVR exposure in the highly desiccation-tolerant mosses *Syntrichia caninervis* and *Syntrichia ruralis*, using a comparative transcriptomics approach. We explored whether UVR protection is physiologically plastic and induced by UVR exposure, addressing the following questions: (1) What is the timeline of changes in the transcriptome with acute UVR exposure in these two species? (2) What genes are involved in the UVR response? and (3) How do the two species differ in their transcriptomic response to UVR? There were remarkable differences between the two species after 10 and 30 min of UVR exposure, including no overlap in significantly differentially abundant transcripts (DATs) after 10 min of UVR exposure and more than twice as many DATs for *S. caninervis* as there were for *S. ruralis*. Photosynthesis-related transcripts were involved in the response of *S. ruralis* to UVR, while membrane-related transcripts were indicated in the response of *S. caninervis*. In both species, transcripts involved in oxidative stress and those important for desiccation tolerance (such as late embryogenesis abundant genes and early light-inducible protein genes) were involved in response to UVR, suggesting possible roles in UVR tolerance and cross-talk with desiccation tolerance in these species. The results of this study suggest potential UVR-induced responses that may have roles outside of UVR tolerance, and that the response to URV is different in these two species, perhaps a reflection of adaptation to different environmental conditions.

## Introduction

Drastic environmental challenges accompanied evolutionary transitions to terrestrial life (Gray, 1993). Low water availability and high solar radiation, including light in the ultraviolet range, would have been major limiting factors for land colonization (Waters, 2003; Becker and Marin, 2009). The first cyanobacteria to colonize land did not fully do so until after the formation of an ozone layer, reducing the amount of ultraviolet radiation (UVR) overall and filtering out the high energy UV-C (Garcia-Pichel, 1998). Tolerance of UVR and desiccation are thought to be ancestral to land plants (Graham et al., 2000, 2004) and, thus, the potential for these traits can be found on all branches of the embryophyte tree of life. For example, the metabolic pathways leading to lignins and flavonoids (two types of molecules important for protection from UVR) likely evolved from ancestral elements of primary metabolism in charophycean algae (Kenrick and Crane, 1997).

Ultraviolet radiation can be damaging to plants—including to important components of plant metabolism like chloroplast membranes, photosystems, and DNA—both via direct absorption and indirectly via reactive oxygen species (ROS). ROS damage other sensitive molecular machinery as well (Apel and Hirt, 2004), and both low and high UV-B doses can generate ROS (Hideg et al., 2013). Tolerance of ROS-generating stressors often exhibit cross-tolerance, where protection from one stressor confers protection for another (Sinclair et al., 2013; Perez and Brown, 2014). For example, in some plants UVR induces the accumulation of transcripts encoding early light-inducible proteins (ELIPs; Singh et al., 2014), which function in both photoprotection and desiccation tolerance in resurrection plants by binding to and protecting photosynthetic pigments (Adamska et al., 1999; Zeng, 2002; Hutin et al., 2003; Oliver et al., 2004; Van Buren et al., 2019). Similarly, transcripts encoding the hydrophilic late embryogenesis abundant (LEA) proteins accumulate under various abiotic stresses in vegetative tissues of plants, including desiccation (Amara et al., 2014; Oliver et al., 2020) where they are thought to help transform cell cytoplasm into the protective biological “glassy state” (Buitink and Leprince, 2004).

If UVR is a stressor, a trade-off to maximize absorption of sunlight but minimize UVR may exist. However, there is increasing evidence that ROS themselves can act as signaling molecules (Apel and Hirt, 2004; Foyer and Noctor, 2009; Triantaphylidès and Havaux, 2009; Mittler et al., 2011; Ray et al., 2012; Hideg et al., 2013; Foyer, 2018). Recently there has been a paradigm shift in understanding UVR as a regulatory signal rather than solely a stressor, as UVR perception is involved in critical metabolic functions (Hideg et al., 2013; Williamson et al., 2014; Neugart and Schreiner, 2018). Still, the distinction between stressor and regulatory signal is not well defined as it is clear that in some cases stress is necessary for acclimation and protection from future stressors (Robson et al., 2019). Recently researchers have begun to classify UVR as a “eustress” (Hideg et al., 2013). In this framework, UV-B is understood to stimulate an alert state that includes defense activation, especially if the radiation is experienced in small doses. Overall, there is increasing evidence and awareness that both UV-A and UV-B can have beneficial effects for plants (Kataria and Guruprasad, 2012; Schreiner et al., 2012; Verdaguer et al., 2017).

Many plants are tolerant of UVR, but there is a wide variation in level and mechanism of tolerance (Boelen et al., 2006). For example, the moss *Physcomitrium* (formerly *Physcomitrella*) *patens* is more UV-B tolerant than *Arabidopsis thaliana*, despite its simpler morphology (Wolf et al., 2010). In fact, nearly all mosses tested in natural settings appear to be minimally damaged by ambient UVR levels (Boelen et al., 2006). Furthermore, in some species UVR protection appears to be physiologically constitutive while in others it is plastic. For example, the Antarctic mosses *Ceratodon purpureus* and *Bryum subrotundifolium* exhibit sun forms that are tolerant of UVR and shade forms that are not but that can be acclimated to UVR within a week in sunlight (Green et al., 2005). On the other hand, in the mosses *Sanionia uncinata, Chorisodontium aciphyllum, Warnstorfia sarmentosa*, and *Polytrichum strictum*, also from Antarctica, UV-B absorbing compounds are not induced by enhanced UV-B radiation (Boelen et al., 2006). Our understanding of the molecular processes that underlie this tolerance, and the extent to which these processes are shared among land plants, remains limited, due in part to the lack of molecular studies in diverse lineages.

*Syntrichia caninervis* and *Syntrichia ruralis* are highly desiccation-tolerant mosses; they can lose almost all of their cellular water and recover after rehydration (Proctor et al., 2007; Wood, 2007). Based on their occurrence in open, exposed habitats (Mishler and Oliver, 1991; Oliver et al., 1993; Bowker et al., 2000), they are presumed to be UVR tolerant (UVT), too. In fact, wild-grown *S. ruralis* and *S. caninervis* plants are unaffected by UV-B radiation, based on chlorophyll fluorescence (Takács et al., 1999; Csintalan et al., 2001; Ekwealor et al., 2021). *Syntrichia ruralis* occupies a wide range of elevations and aridities ranging from arid to mesic (Oliver et al., 1993). Phylogenetic and taxonomic delineations within the *S. ruralis* complex are a topic of active investigation, but for the purposes of this study it is only important to note that the genotype of *S. ruralis* used here was from a relatively mesic habitat so can be expected to be adapted to mesic conditions. *Syntrichia caninervis*, on the other hand, is common in low elevation arid environments where it experiences frequent and prolonged desiccation (Oliver et al., 1993), and the genotype used here was from a relatively xeric habitat.

Mosses exposed to UVR while hydrated may respond with different mechanisms of protection than the passive responses that would be necessary in a desiccated plant. Specifically, mesic-adapted plants may utilize a more active response to UVR, such as ROS scavenging (Cooper-Driver et al., 1998; Grace and Logan, 2000; Clé et al., 2008; Rustioni, 2017), while arid-adapted species may have passive protection, such as UVR-absorbing compounds, or sunscreens (Taipale and Huttunen, 2002; Newsham, 2003; Robinson et al., 2005; Clarke and Robinson, 2008; Robinson and Waterman, 2014; Waterman et al., 2017). Mosses that live in mesic habitats like *S. ruralis* may experience prolonged periods of intense UVR while hydrated, such as after a summer rain. In contrast, in its natural dryland habitat, *S. caninervis* experiences prolonged periods of high levels of UVR while quiescent, and overcast conditions while hydrated (Mishler and Oliver, 1991; Marschall and Proctor, 2004).

The evolution of physiological ecology in *Syntrichia*, including the balance of local genetic adaptation relative to physiological plasticity in response to different environmental conditions, is a topic for future studies. Here, we set the stage by investigating the genetic underpinnings of response to acute broadband UVR exposure in *S. caninervis* and *S. ruralis*, using a comparative transcriptomics approach. In particular, we aimed to uncover whether UVR protection is physiologically plastic and induced by UVR exposure by asking the following questions: (1) What is the timeline of changes in the transcriptome with acute UVR exposure in these two species; (2) What genes are involved in the acute UVR response; and (3) How do the two species differ in their transcriptomic response to UVR?

## Materials and Methods

### Experimental Conditions

To compare effects of acute UVR exposure on *S. caninervis* and *S. ruralis*, we grew isolates of both in a single white fluorescent light environment with no UVR. Shoots from an isolated clone of *S. caninervis* from southern Nevada, United States (*Stark NV-107*, United States, Nevada, Clark County, Newberry Mts, Christmas Tree Pass; UNLV) and an isolated clone of *S. ruralis* from Calgary, Alberta, Canada (*Brinda 9108*, Canada, Calgary, Bow River; UNLV) were cultivated in a growth chamber set to an 18-h photoperiod (18°C light and 8°C dark), at ca. 30 μmol m^–2^ s^–1^ PAR. The light source was 24″ F20 T12 GE Plant and Aquarium bulbs, warm tone, 3100 K, producing 750 lumens (GE Lighting, Boston, MA, United States). Cultures of a single genotype for each species were grown in lidded approximately 77 mm × 77 mm × 97 mm Magenta GA-7 plant culture boxes (bioWORLD, Dublin, OH, United States) from fragments on 1.2% agar made with an inorganic nutrient solution (Hoagland and Arnon, 1950).

After five months of growth without UVR exposure, the mature plants were subjected to a UVR exposure time series in triplicate. First, in order to filter out UV-C radiation, which is not present in solar radiation that reaches earth but may be in artificial UVR sources, culture box lids were replaced with 7.6 cm × 7.6 cm (3″ × 3″) acrylic windows, 3.175 mm (1/8 in) thick (Polycast Solacryl SUVT acrylic, Spartech, Maryland Heights, MO, United States), sealed to the culture boxes with wax film. These UV-transmitting windows transmit at least 90% across the visible and UV-A/B spectrum and then drop to near 0% transmittance near the boundary of UV-C (between 275 nm and 250 nm)^1^. The sides of each culture box were wrapped in aluminum foil to ensure all light reaching plant cultures passed through the installed window. Prepared culture boxes were placed under four T8 reptile bulbs (ReptiSun 10.0 UVB, Zoo Med Laboratories Inc., San Luis Obispo, CA, United States).

Treatment light environment consisted of ca. 36 μmol m^–2^ s^–1^ broadband UV-AB radiation with a UV-B fluence rate of 0.36 mW cm^–2^ and ca. 120 μmol m^–2^ s^–1^ PAR. This UV-B radiation level was selected to be within the approximate range of global irradiance (Pinedo et al., 2006), with intention to expose plants to a realistic level of UV-B radiation while ensuring the dose was sufficiently high to be detected by the plants. Broadband UVR and PAR were measured with LightScout UV and Quantum Sensors and the LightScout Sensor Reader (Spectrum Technologies, Aurora, IL, United States) while UV-B fluence was measured at several locations under the lamps with a handheld radiometer that was last calibrated in 2014 and independently evaluated in 2016 (SKU 430, Apollo Display Meter, Skye Instruments Ltd., Llandrindod Wells, United Kingdom). All sensors were covered with the same UV-transmitting acrylic as the samples prior to measurements and matched to the distances between plant, window, and light source. The first tissue collection was made after 10 min of exposure (T_10_) and 30 min for the second (T_30_). At the same time, control samples that were never subjected to a UVR treatment were collected (T_0_). At collection, tissues were quickly snipped at the base in an effort to collect above-ground tissues and minimize agar collection, placed into 1.6 mL microcentrifuge tubes with a push-pin hole in the top, flash-frozen in liquid nitrogen, and stored at −80°C until further processing.

### Extraction and Sequencing

Frozen tissue was sent to Novogene (Novogene, Sacramento, CA, United States) for RNA extraction, library preparation, and transcriptome sequencing. There were three replicates made for each of the three treatments for both species, yielding 18 samples total. Total RNA was extracted with the Zymo *Quick*-RNA Plant Kit (Zymo Research, Irvine, CA, United States). RNA samples were processed according to standard Novogene protocols, including preliminary quality checks with gel electrophoresis followed by quantification and purity assessment with NanoDrop (Thermo Fisher Scientific, Waltham, MA, United States), and sample integrity assays with a Bioanalyzer 2100 (Agilent, Santa Clara, CA, United States). All 18 samples had an RNA integrity (RIN) score of at least 7. After quality checking procedures, oligo(dT) beads were used to enrich eukaryotic mRNA and rRNA was removed with the Illumina Ribo-Zero kit (Illumina, Inc., San Diego, CA, United States). RNA samples were then reverse-transcribed into double-stranded cDNA libraries by randomly fragmenting mRNA with fragmentation buffer, followed by adding random hexamers primer, a custom second-strand synthesis buffer (Illumina, Inc., San Diego, CA, United States), dNTPs, RNase H, and DNA polymerase I to initiate the second-strand synthesis. Next, after terminal repair and adaptor ligation, library preparation was completed with size selection and PCR enrichment. Library quality was assessed with Qubit 2.0 (Thermo Fisher Scientific, Waltham, MA, United States) to test preliminary library concentration, a Bioanalyzer 2100 (Agilent, Santa Clara, CA, United States) to test the insert size, and quantitative PCR to precisely quantify the library effective concentration size. Finally, libraries were sequenced on the 150 bp paired-end Illumina NovaSeq 6000 platform (Illumina, Inc., San Diego, CA, United States).

### Transcriptome Assembly

Transcriptomic data were first cleaned with Trimmomatic version 0.39 (Bolger et al., 2014) using a sliding window of four base pairs with a Phred quality score cutoff of 20, a minimum length of 20, and with a leading and trailing minimum of three. Bowtie2 (Langmead and Salzberg, 2012) and Tophat2 (Kim et al., 2013) were used to make indexes of the reference *S. caninervis* genome (Silva et al., 2020) for mapping and assembly. If multiple isoforms of a gene were detected in the transcriptome, they were binned and counted per parent gene. Transcripts with fewer than 10 read counts were discarded before downstream analyses. To account for overdispersion, transcript counts were transformed with the variance-stabilizing transformation (VST) prior to visualization with principal components analyses (PCAs) on all 18 samples together as well as on the nine samples from each species separately.

### Differential Transcript Abundance

In order to quantify transcripts involved in the acute UVR response in *S. ruralis* and *S. caninervis*, reads were first mapped to the reference *S. caninervis* genome (Silva et al., 2020) with Tophat2 (Kim et al., 2013). Htseq-count version 0.9.1 (Anders et al., 2015) was used to estimate read counts per sample per gene and final analyses were performed in R (R Core Team, 2019) using DESeq2 (Love et al., 2014) to test for differential transcript abundance in plants at each point in the UVR treatment time series in each species. To identify sets of significant transcripts, *P*-values were first corrected with the Benjamini-Hochberg correction (Benjamini and Hochberg, 1995) to account for the false discovery rate (FDR) of multiple tests (Jafari and Ansari-Pour, 2019), followed by an adjusted *P*-value (*P*-adj) cut-off of 0.05 and an absolute value of the log2-fold change (LFC) minimum of 1. Sets of significantly increased or decreased transcripts at each time point in each species were compared and checked for overlap. Additionally, to test if distributions of Gene Ontology (GO; Gene Ontology Consortium, 2004) functional annotations were different for each set of significantly abundant transcripts from the overall pool of GO terms in the transcriptome, Fisher’s exact tests were performed in R for each of the GO categories (Biological Processes, Molecular Functions, and Cellular Components) using a modified version of the protocol described by Tribble et al. (2021). Additionally, individual GO terms that were enriched in each significantly differentially abundant transcript set relative to the whole transcriptome were detected using a maximum likelihood framework based on a binomial distribution as in Tribble et al. (2021). The script was modified to adjust *P*-values with the Benjamini-Hochberg FDR correction. Annotations were derived from the *S. caninervis* genome (Silva et al., 2020) and when annotations for multiple isoforms existed in the reference, the annotations (including GO terms) of the longest isoform were used.

### Comparison of Abundance Patterns Across Species

Samples from the two study species were also analyzed together in a likelihood ratio test (LRT) with the reduced model of ∼species + time and a *P*-adj cut-off of 0.05 to test for transcripts that have different transcript abundance changes in the two species over the UVR treatment time course, controlling for differences at T_0_. Next, VST transformed transcript counts from genes whose transcript abundance pattern differed significantly between the two species, identified via the LRT, were used in a cluster analysis to identify groups of genes that have similar patterns using the R package DEGreport (Pantano, 2020), with a minimum of five genes per cluster enforced. Transcript abundance Z-scores were calculated to scale and average replicates for plotting visualization.

### Candidate Gene Families and Functions

All sets of significant transcripts were checked for the presence of two candidate gene families that were identified *a priori*: transcripts encoding early light-inducible proteins (ELIPs) and transcripts of late embryogenesis abundant genes (LEAs), using orthogroup lists in Silva et al. (2020). Both ELIPs and LEAs are involved in vegetative desiccation tolerance in *S. caninervis* as well as other resurrection plants (Costa et al., 2017; Van Buren et al., 2019; Silva et al., 2020) and were selected for the potential for cross-talk and cross-tolerance between desiccation tolerance and UVT. Furthermore, ELIPs function in photoprotection and in some plants the accumulation of ELIP transcripts is mediated by UVR8, the specialized UV-B receptor 8 (Hutin et al., 2003). Additionally, ELIPs, LEAs, and other genes with specific GO terms including: *photosynthesis*, *response to light stimulus*, *response to stress*, and *photomorphogenesis* were tested for differential transcript abundance as a group, relative to the distribution of differential abundance of all transcripts, following a modified version of the procedure described in Tribble et al. (2021). In brief, for each group of *n* genes (e.g., ELIPs), *n* transcripts were randomly sampled 10,000 from the pool of all assayed transcripts. The absolute LFC values of these sets of 10,000 samples were then compared to the distribution of absolute-value LFCs of the full assayed transcriptome and effect sizes were estimated with the Mann-Whitney *U* test (Mann and Whitney, 1947). Furthermore, number of significantly differentially abundant transcripts (DATs) after 10 and 30 min of UVR exposure in each species and in each gene group were tested against the number of significant transcripts in the 10,000 random samples to test if there were more or fewer than expected by chance.

### Code

The following libraries and tools were also used for analysis and visualization: tidyverse (Wickham et al., 2019), dplyr (Wickham et al., 2020), VennDiagram (Chen and Boutros, 2011), ggplot2 (Wickham, 2016), and the scripts in https://github.com/cmt2/bomTubers. Analysis code for this project is available on GitHub^2^.

## Results

### Transcriptome Assembly and Assessment

After quality filtering, a total of 905 M reads were recovered for all 18 samples; an average of about 50 million reads each. *Syntrichia ruralis* samples had an average read mapping rate of 51.2% while *S. caninervis* samples had an average read mapping rate 63.1% (per-sample mapping rates in Supplementary Table 1). In total, 6,851 genes were recovered in the transcriptome of *S. ruralis* and 6,868 genes in that of *S. caninervis.*

**FIGURE 1:**
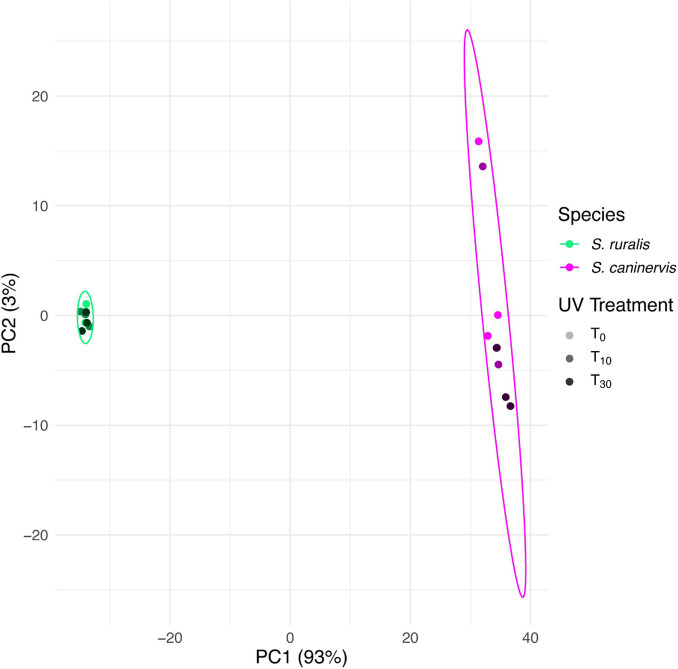
Principal components biplot of 1st and 2nd PCA scores based on transcript abundance in *Syntrichia ruralis* and *S. caninervis* with 0, 10, or 30 min of UV radiation exposure. Multivariate normal distribution 95% data ellipses were drawn for each species. Transcriptomes were prepared in triplicate.

On the PCA of VST transcript counts of both species (*Syntrichia ruralis* and *S. caninervis*) at all three UVR treatment time points (T_0_, T_10_, and T_30_), the two species separated strongly along the first PCA axis, which explained 93% of the variation (Figure 1). *Syntrichia ruralis* was tightly clustered while *S. caninervis* spread along PC2, which explained just 3% of the variation. There was no strong pattern of separation of UVR treatments on these two axes of the PCA, although UVR treatment did somewhat separate along PC2, but mostly due to variation among replicates within treatments.

**FIGURE 2:**
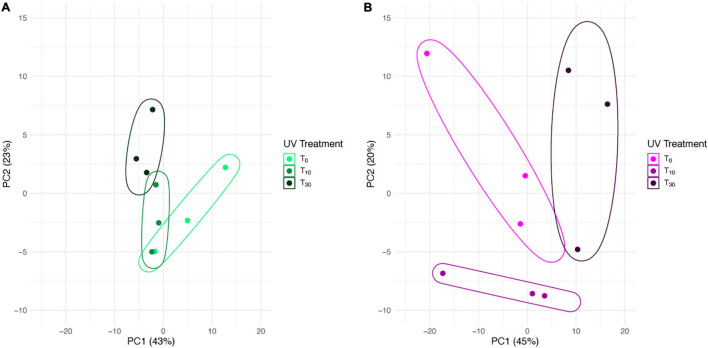
Principal components biplot of 1st and 2nd PCA scores based on transcript abundance in *Syntrichia* with 0, 10, or 30 min of UV radiation exposure. Enclosing data ellipses were estimated using the Khachiyan algorithm (Khachiyan, 1979). Transcriptomes were prepared in triplicate. **(A)** *Syntrichia ruralis.* **(B)** *Syntrichia caninervis.*

In the PCA for *S. ruralis* alone, the three treatment groups separated slightly along axis PC1, which explained 43% of the variation (Figure 2A). However, T_0_ spread widely across this axis and overlapped with T_10_. There was some separation of T_10_ and T_30_ along PC2, which explained 23% of the variation, though again T_0_ spread widely across this axis, especially in the same range as T_10_. In the PCA of *S. caninervis* alone, PC1, which explained 45% of the variation, separated T_30_ from the other two treatments (T_0_ and T_10_), though with some overlap with T_0_. T_0_ and T_10_ were separated along PC2, which explained 20% of the variation (Figure 2B).

### Differential Transcript Abundance

**FIGURE 3:**
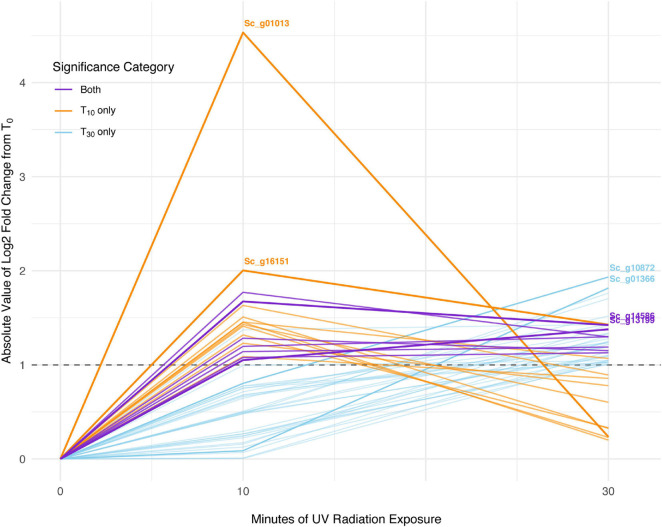
Absolute-value log2-fold change of transcripts significant with 10 min of UV radiation, 30 min of UV radiation, and with both treatments in *Syntrichia ruralis.* Dashed line shows log2-fold change (LFC) of 1, the significance cut-off used for these transcript sets. The transcripts with the largest absolute-value LFC in each category are labeled with their gene identification. Labeled genes are: Sc_g01013, ribulose bisphosphate carboxylase small chain clone 512-like; Sc_g16151, uncharacterized protein LOC112291873; Sc_g10872, heavy metal-associated isoprenylated plant protein 28-like; Sc_g01366, uncharacterized protein LOC112287812; Sc_g14586, pectinesterase 2-like; and Sc_g13199, probable sodium/metabolite cotransporter BASS3, chloroplastic.

In *S. ruralis*, 18 transcripts were significantly differentially abundant between T_0_ (no UVR exposure) and T_10_ (10 min of UVR exposure), none of which were ELIPs or LEAs (Supplementary Table 2). Between T_0_ and T_30_, there were 38 transcripts significantly differentially abundant, which included two LEAs but no ELIPs (Supplementary Table 3). Seven individual transcripts were significantly differentially abundant in both the T_0_ versus T_10_ and the T_0_ versus T_30_ sets (Figure 3). Of the three GO categories (Biological Processes, Molecular Functions, and Cellular Components) in the significant transcript sets for T_10_ and T_30_ (30 min of UVR exposure) for *S. ruralis*, only those for Molecular Functions in transcripts significantly different at T_10_ were significantly different from the overall distribution of GO terms (*P* = 0.0003).

**FIGURE 4:**
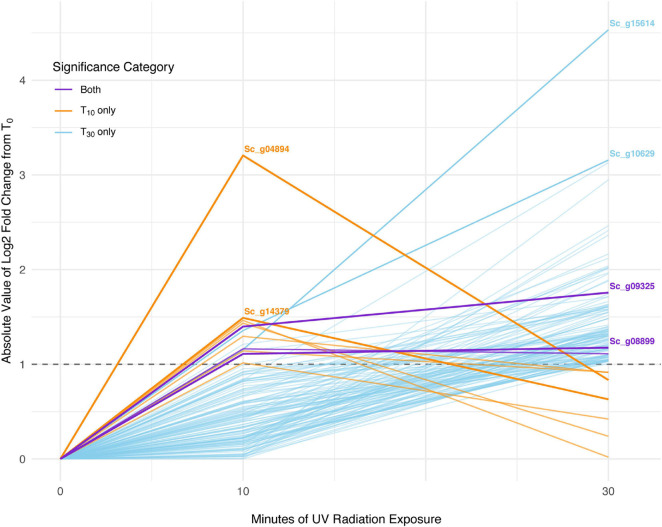
Absolute-value log2-fold change of transcripts significant with 10 min of UV radiation, 30 min of UV radiation, and with both treatments in *Syntrichia caninervis.* Dashed line shows log2-fold change (LFC) of 1, the significance cut-off used for these transcript sets. The transcripts with the largest absolute-value LFC in each category are labeled with their gene identification. Labeled genes are: Sc_g04894, uncharacterized protein LOC112281062; Sc_g14379, poly(ADP-ribose) polymerase 3; Sc_g15614, lachrymatory-factor synthase; Sc_g10629, hypothetical protein PHYPA_004803; Sc_g09325, chloroplastic early light-induced protein; and Sc_g08899, protein EARLY-RESPONSIVE TO DEHYDRATION 7, chloroplastic-like isoform X2.

In *S. caninervis*, of the 6,868 genes detected in the transcriptome, 10 were differentially abundant between T_0_ and T_10_, one of which was an ELIP and none of which were LEAs (Supplementary Table 4). When comparing T_0_ and T_30_, there were 126 genes that were significantly differentially abundant, which contained two ELIPs and four LEAs (Supplementary Table 5). Three transcripts were significantly differentially abundant in both the T_0_ versus T_10_ and the T_0_ versus T_30_ sets (Figure 4). Distribution of GO terms was not significantly different in any of the GO categories for significant transcript sets for T_10_ or T_30_. One GO term, GO:0016021 *integral component of membrane*, was significantly more abundant in the significant transcript set than in the overall transcriptome (*P* = 0.043).

**FIGURE 5:**
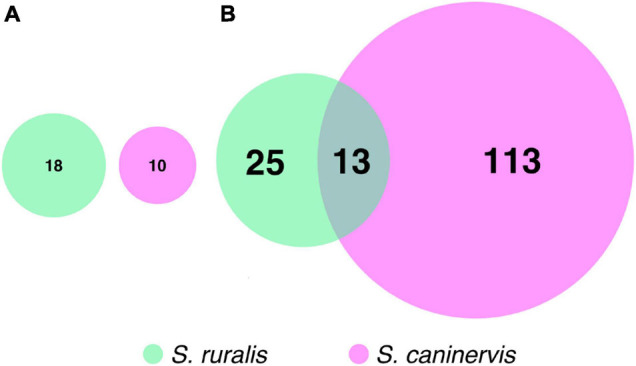
Euler’s diagrams of differentially abundant transcripts with **(A)** 10 min and **(B)** 30 min of UV radiation exposure in *Syntrichia ruralis* and *S. caninervis.*

While 18 transcripts were significantly differentially abundant at T_0_ in *S. ruralis* and 10 were in *S. caninervis*, none of these were shared between the two (Figure 5A). In contrast, there were many more DATs in *S. caninervis* (126) than in *S. ruralis* (38) at T_30_, and 13 transcripts were significant in both (Supplementary Table 6 and Figure 5B).

**FIGURE 6:**
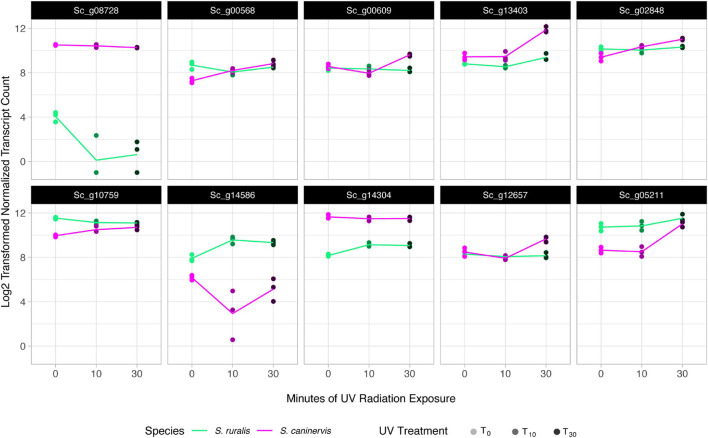
Transcript abundance in the top 10 most significant species-specific response transcripts to 10 and 30 min of UV radiation exposure in *Syntrichia ruralis* and *S. caninervis*. Genes shown are: Sc_g08728, Core-2/I-branching beta-16-N-acetylglucosaminyltransferase family protein; Sc_g00568, putative 12-oxophytodienoate reductase 11; Sc_g00609, adenine phosphoribosyltransferase; Sc_g13403, uncharacterized protein LOC112285453 isoform X2; Sc_g02848, protein PROTON GRADIENT REGULATION 5, chloroplastic; Sc_g10759, NPC intracellular cholesterol transporter 1-like; Sc_g14586, pectinesterase 2-like; Sc_g14304, hypothetical protein MARPO_0010s0178; Sc_g12657, uncharacterized protein LOC112283607; and Sc_g05211, treponemal membrane protein B-like isoform X1.

**FIGURE 7:**
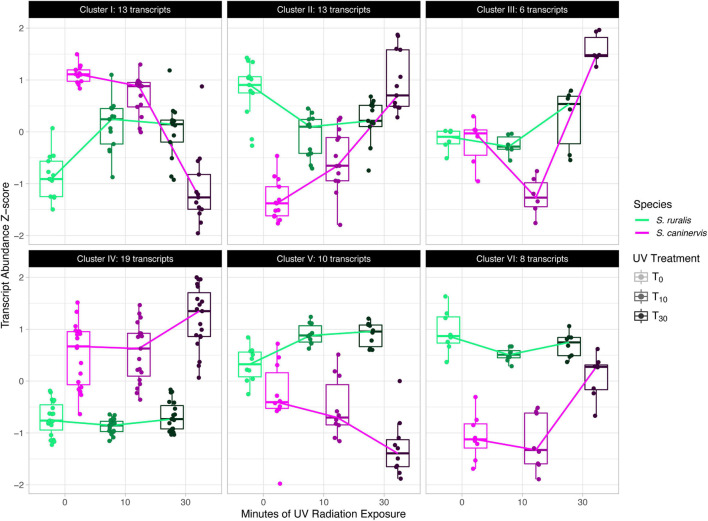
Cluster analysis of significant species-specific response transcripts to 10 and 30 min of UV radiation exposure in *Syntrichia ruralis* and *S. caninervis.*

When analyzed together for a likelihood ratio test to identify transcripts that have a different abundance pattern at T_10_ and T_30_ while controlling for differences at T_0_, a total of 6,859 transcripts were assayed. Of these, 69 were significantly different in the two species (Supplementary Table 7 and Figure 6). Cluster analyses of these transcripts identified six transcript clusters, each containing 6–19 transcripts (Supplementary Table 8 and Figure 7).

### Candidate Gene Families and Functions

**FIGURE 8:**
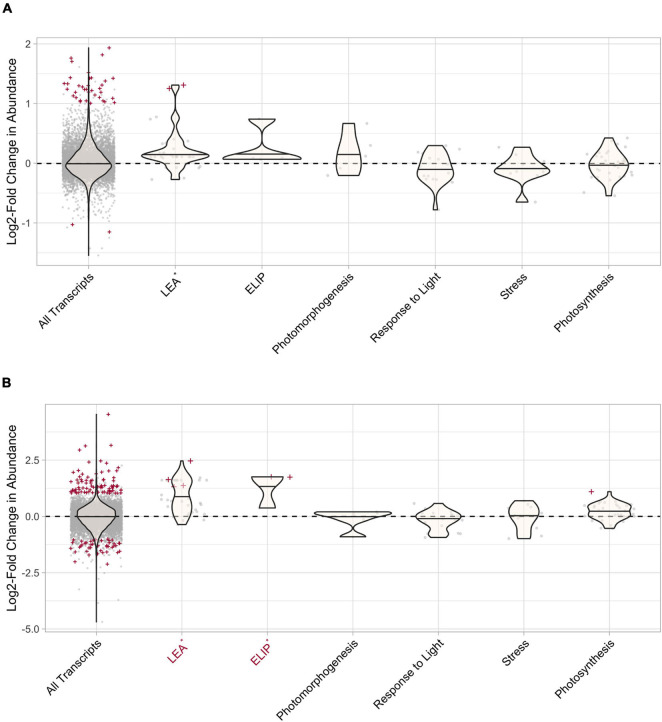
Differential transcript abundance of candidate gene groups after 30 min of UV radiation exposure in **(A)** *Syntrichia ruralis* and **(B)** *Syntrichia caninervis.* Positive values correspond to increased transcript abundance after 30 min of UV radiation treatment, negative values correspond to decreased transcript abundance. Violin plots represent the log2-fold change (LFC) value for the gene groups and gray points correspond to LFC of individual transcripts. Transcripts that are significantly differentially abundant (*P*-adj < 0.05, absolute-value LFC > 1) are labeled with red crosses and groups with absolute-value LFC distributions that are significantly larger than that of the whole transcriptome (All Transcripts) are labeled in red on the *x*-axis. Groups with significantly more differentially abundant transcripts than expected by change are labeled with an asterisk on the *x*-axis.

In *S. ruralis*, none of the hypothesized candidate groups had a significantly higher distribution of absolute-value LFCs than the entire transcriptome at T_30_ (Supplementary Figure 1 and Figure 8A). One candidate group, LEAs, had more DATs at T_30_ than expected by chance (Supplementary Figure 2 and Figure 8A). At T_10_, ELIPs, LEAs, and transcripts with the GO term *photosynthesis* had a significantly higher distribution of absolute-value LFCs than the entire transcriptome (Supplementary Figures 3, 4) and transcripts with the GO term *photosynthesis* had more DATs than expected by chance (Supplementary Figure 5).

In *S. caninervis*, both LEAs and ELIPs had a significantly higher distribution of absolute-value LFCs than the entire transcriptome at T_30_ (Supplementary Figure 6 and Figure 8B) and had more DATs than expected by chance (Supplementary Figure 7 and Figure 8B). At T_10_, ELIPs had a significantly higher distribution of absolute-value LFCs than the entire transcriptome (Supplementary Figures 8, 9) and more DATs than expected by chance (Supplementary Figure 10).

## Discussion

There were strong differences between *S. ruralis* and *S. caninervis* on the scale of the whole transcriptome. In fact, 93% of the variation in the transcriptome was explained by overall species differences, as compared to relatively little effect of UVR treatment (Figure 1). In both species, the number of DATs increased with increasing UVR exposure time. However, there were some striking differences in number of DATs between the two species. For instance, *S. ruralis* had more DATs at T_10_ than *S. caninervis* did (Figure 5A), while *S. caninervis* had many more at T_30_ (Figure 5B). Overall, *S. ruralis* had fewer DATs with UVR treatment than *S. caninervis* did. This finding might suggest that this level of UVR is a more significant stressor for hydrated *S. caninervis* than it is for hydrated *S. ruralis*, supporting the hypothesis that the latter may be adapted to tolerating UVR for prolonged periods while hydrated. While number of transcripts does not indicate magnitude of stress *per se*, large stressors do generally have large effects on transcriptomes. For example, *Physcomitrium patens* is considered more UVR tolerant than *A. thaliana*, based on tissue growth, bleaching, and death (Wolf et al., 2010). Correspondingly, 1 h of narrow-band UV-B radiation resulted in only one differentially abundant transcript in *P. patens* but the same conditions resulted in more than several hundred differentially abundant genes in *A. thaliana* (Favory et al., 2009; Wolf et al., 2010). In fact, both species in this study have relatively low numbers of DATs with UVR exposure, compared to other stressors, which might be reflective of a high level of UVR tolerance, as has been argued for other species and stressors (Richardt et al., 2010; Wolf et al., 2010).

### Differentially Abundant Transcripts With 10 and 30 min of Ultraviolet Radiation

At T_10_, *S. ruralis* had 18 DATs compared to T_0_. Putative functions of those genes included oxidation-reduction via peroxidases, photosynthetic monooxygenases, oxygen- and various metal-binding, and membrane and cell wall modifications (Supplementary Table 2). The differentially abundant transcript with the largest absolute-value LFC was of the gene Sc_g01013 – ribulose bisphosphate carboxylase small chain clone 512-like – a component of ribulose-1,5-bisphosphate carboxylase/oxygenase (RuBisCo), which is essential for photosynthesis and photorespiration (Table 1). This transcript had a log2-fold increase of more than 4.5 with 10 min of UVR exposure. Similarly, Sc_g13199 – probable sodium/metabolite cotransporter BASS3, chloroplastic, an integral component of the chloroplast envelope – was increased. A relationship between UVR and photosynthesis is well-documented; most commonly UV-B radiation-induced impairment of photosynthesis has been observed (Vass, 1997; Sicora et al., 2006). While several components of photosynthetic machinery are susceptible to UVR-induced damage, one clear cause of photosynthetic impairment when exposed to intense or prolonged UVR is degradation of RuBisCo (Jordan et al., 1992; Nogués and Baker, 1995; Bischof et al., 2000). Along with other transcripts involved in oxidation-reduction processes, increased abundance of these transcripts may suggest ROS accumulation, oxidative stress, and photosynthetic machinery degradation from acute UVR exposure in *S. ruralis.* Interestingly, Sc_g01013 – ribulose bisphosphate carboxylase small chain clone 512-like – dropped drastically again at T_30_ (Figure 3), suggesting this is a uniquely early-exposure response.

**TABLE 1:** Putative function and gene ontology (GO) terms for the top 10 most differentially abundant transcripts with 10 min of broadband UVR exposure in *Syntrichia ruralis*.

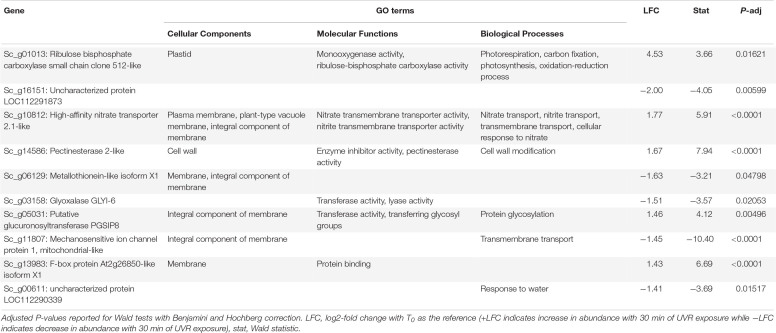

At T_30_ the 38 DATs in *S. ruralis* (Supplementary Table 3) also included increases of several genes involved in oxidation-reduction processes and chloroplastic proteins, suggesting a continued response to UVR-induced ROS in the photosynthetic apparatus. The top-most differentially abundant transcript was Sc_g10872 – heavy metal-associated isoprenylated plant protein (HIPP) 28-like – a metallochaperone responsible for safe transport of metallic ions within the cell (Table 2). In *Oryza sativa*, HIPPs are also involved in cold and drought stress (de Abreu-Neto et al., 2013). Although it did not meet the significance threshold at T_10_, abundance of this transcript increased consistently over the 30-min time series (Figure 3), suggesting a relationship between UVR exposure time and transcript abundance. Similarly, transcripts of Sc_g07931 – senescence/dehydration-associated protein At4g35985, chloroplastic-like, which is involved in cold and salt stress – increased at T_30_. It is possible that in *S. ruralis* these genes are involved in UVR protection or that they are involved in something else entirely but that the response is UVR-induced in a case of genetic cross-talk (Sinclair et al., 2013).

**TABLE 2:** Putative function and gene ontology (GO) terms for the top 10 most differentially abundant transcripts with 30 min of broadband UVR exposure in *Syntrichia ruralis*.

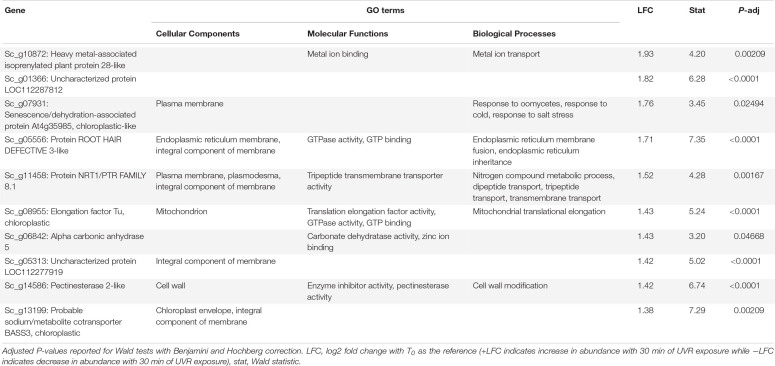

In *S. caninervis*, the 10 transcripts significantly changed in abundance at T_10_ contained several genes involved in the mitochondrial and other membranes (Supplementary Table 4), which are targets of ultraviolet radiation in plants (Murphy, 1983). Along with one significant transcript involved in oxidation-reduction, the high number of membrane-involved transcripts may be a sign of stress or simply an acclimatory response to protect membranes in the new environmental conditions. Indeed, the distinction between stress and regulatory acclimation is not easily defined (Hideg et al., 2013; Robson et al., 2019). The top-most differentially abundant transcript, which decreased with an LFC of −3.2, was Sc_g04894 – uncharacterized protein LOC112281062 – with only one GO term, *response to water* (Table 3). As this protein is uncharacterized, the role it is playing in UVR response can only be speculated upon, but given the high level of desiccation tolerance in this species, the functional annotation is intriguing. In fact, UVR has been implicated in desiccation tolerance in *S. caninervis*, where removal of natural levels of UVR from a field setting hindered recovery of photosynthetic efficiency from desiccation (Ekwealor et al., 2021). By T_30_, abundance of this transcript had increased again to nearly an LFC of −1 (Figure 4).

**TABLE 3:** Putative function and gene ontology (GO) terms for top 10 most differentially abundant transcripts with 10 min of broadband UVR exposure in *Syntrichia caninervis.*

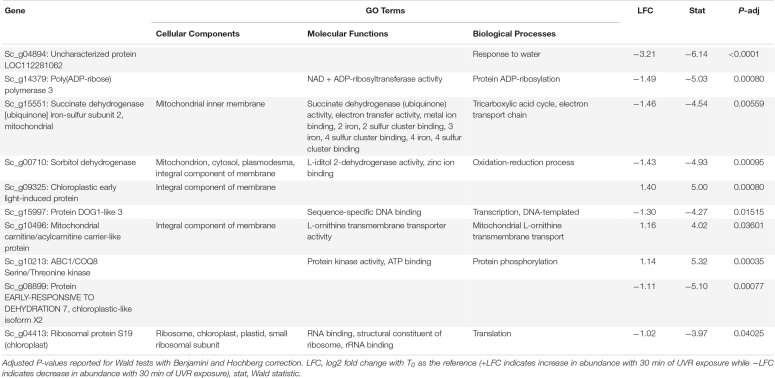

Interestingly, many of the 126 transcripts differentially abundant for *S. caninervis* at T_30_ were also for uncharacterized proteins (Supplementary Table 5 and Table 4). Many of these increased consistently with increased UVR exposure, though some absolute-value LFCs remained low at T_10_ and increased in abundance only at T_30_ (Figure 4). Furthermore, several of these transcripts were involved in oxidation-reduction and in membranes. Indeed, the GO term *integral component of membrane* was enriched in the DATs, relative to the transcriptome. UV-B radiation-induced ROS can cause oxidative damage to lipids, leading to lipid peroxidation, membrane permeability, and disruption of membrane integrity (Foyer et al., 1994; Dai et al., 1997; Alexieva et al., 2001). While the functions of these uncharacterized genes are not known, they may be involved in either protection or repair of cell or thylakoid membranes.

**TABLE 4:** Putative function and gene ontology (GO) terms for top 10 most differentially abundant transcripts with 30 min of broadband UVR exposure in *Syntrichia caninervis.*

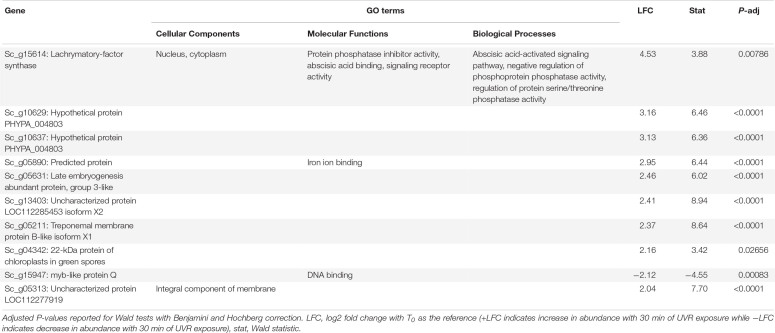

### Candidate Gene Families and Cross-Talk With Desiccation

*Syntrichia caninervis* had one and two differentially abundant ELIP transcripts at T_0_ and T_30_, respectively, while *S. ruralis* had none at either time point. Additionally, the entire set of ELIPs was significantly increased and had a higher number of significantly differentially abundant ELIPs than expected by chance at T_30_, suggesting a strong ELIP response with 30 min of UVR exposure in *S. caninervis*. Increased abundance with UVR may be due to perception of UV-B radiation in particular (Singh et al., 2014). Plants perceive UV-B radiation via the specialized UV-B receptor 8 (UVR8; Ulm and Nagy, 2005), which is remarkably conserved in structure and mechanism across land plants and green algae (Ulm and Nagy, 2005; Tilbrook et al., 2013; Jenkins, 2014). However, the regulatory role that UVR and the UVR8 receptor play may not be conserved (Robson et al., 2015). For example, a cytochrome P450 monooxygenase 98 (CYP98) enzyme is involved in biosynthesis of UV-absorbing pigments in some plants (Ehlting et al., 2006; Nelson and Werck-Reichhart, 2011), including phenolic components of the cuticle of *P. patens*, but acts in the first irreversible step in the biosynthesis of lignin in tracheophytes (Renault et al., 2017). In some plants UVR8 mediates the accumulation of transcripts encoding early light-inducible proteins (ELIPs; Singh et al., 2014), which function in photoprotection (Hutin et al., 2003) and desiccation tolerance in resurrection plants (Zeng, 2002; Oliver et al., 2004; Van Buren et al., 2019). While it is not yet known if or how ELIPs might be involved in UVT, their role in other forms of photoprotection and in desiccation tolerance suggest that this may also be an example of cross-talk in underlying pathways (Zeng, 2002; Hutin et al., 2003; Oliver et al., 2004; Van Buren et al., 2019).

In both *S. ruralis* and *S. caninervis*, late embryogenesis abundant (LEA) genes were more significantly differentially abundant at T_30_ than expected by chance, while no LEAs were significantly differentially abundant at T_0_ in either species. LEAs are involved in desiccation tolerance in *S. caninervis* as well as other resurrection plants (Costa et al., 2017; Van Buren et al., 2019; Silva et al., 2020). Moreover, some LEAs specifically function in protection against oxidative stress from ROS, even outside of drought conditions (Mowla et al., 2006). Both species had one significant group 3 LEA and one D-29 type (Sc_g05631 – late embryogenesis abundant protein, group 3-like, and Sc_g13874 – late embryogenesis abundant protein D-29), while *S. caninervis* additionally had a second group 3 type (Sc_g00779 – late embryogenesis abundant protein, group 3-like) and an additional group 14 LEA (Sc_g10451 – putative late embryogenesis abundant protein, LEA-14). Furthermore, the entire set of LEAs in *S. caninervis* were more differentially abundant than expected by chance at T_30_. Given the role of LEAs in desiccation tolerance, these differences may reflect adaptations to different habitats where prolonged UVR is experienced in different hydration states.

### Species-Specific Transcriptomic Responses

Of the top 10 most species-specific transcript abundance responses with UVR exposure, two in particular had striking differences in response to UVR over the time series (Figure 6 and Table 4). The first of which, Sc_g08728 – Core-2/I-branching β-16-N-acetylglucosaminyltransferase family protein – is involved in biosynthesis of asparagine-attached glycans (*N*-glycans), which function in stress tolerance in *A. thaliana* (von Schaewen et al., 2008). This gene appears to be constitutively expressed in *S. caninervis* and remains in high abundance with or without UVR treatment, while abundance in *S. ruralis* is much lower with no UVR and drops drastically with UVR exposure. Sc_g14586 – pectinesterase 2-like – also shows an interesting pattern with UVR treatment in the two species. Pectinesterases are cell-wall-associated enzymes that have been implicated in many components of plant development (Phan et al., 2007). Transcript abundance is similar in the two species prior to UVR exposure, though higher in *S. ruralis*, but they respond to UVR treatment in opposite ways. In *S. ruralis*, transcript abundance increases at 10 min and decreases again slightly at 30 min. In *S. caninervis*, abundance decreases drastically then increases again. It is unclear what this gene could be doing in response to UVR, if anything, but this pattern nonetheless again demonstrates that these two species have different responses.

Six clusters of transcripts that have significantly different responses to 10 and 30 min of UVR exposure in the two species after controlling for differences at T_0_ were identified (Supplementary Table 8). Cluster I, containing 13 transcripts, is characterized by an increase in abundance at T_10_ and a plateau at T_30_ in *S. ruralis*, but a decrease with increasing UVR time in *S. caninervis* (Figure 7). Many of these thirteen transcripts are for genes involved in the nucleus and in nucleic acid or protein binding. In fact, only nine of the thirteen genes have GO annotations at all, and six of them have at least one of the following GO Molecular Functions: *nucleic acid binding*, *protein binding*, *DNA-binding transcription factor activity*, and *DNA binding*; or a Cellular Components annotation of *nucleus.* Cluster V has a similar pattern in the two species and also has several GO annotations involving DNA binding and nuclear location. DNA absorbs wavelengths in the ultraviolet spectrum which can cause DNA breaks and lesions, and plants may respond to UVR-induced damage with DNA binding for repair (Pang and Hays, 1991; Tuteja et al., 2009; Gill et al., 2015). These patterns suggest the possibility that *S. ruralis* and *S. caninervis* may respond differently in terms of DNA protection when exposed to UVR. Cluster VI also has interesting patterns in the two species: high abundance with a small decrease at T_10_ in *S. ruralis*, lower abundance with a large increase at T_30_ in *S. caninervis*. Of the eight transcripts in this cluster, seven of them have GO annotations and all of them have a Cellular Components annotation of *integral component of membrane*, *membrane*, *plasma membrane*, or some combination of these. This may suggest some constitutive level of membrane-related transcript abundance in *S. ruralis* while *S. caninervis* responds with membrane-related transcripts only with increasing UVR exposure.

### Summary

Mosses exposed to UVR while hydrated may utilize a more active response to UVR, while arid-adapted species may have passive protection. In this study we found little overlap in the transcriptomic response to broadband UVR between xeric *S. caninervis* and the more mesic *S. ruralis.* One explanation for the wide distribution of *S. ruralis* could be that it has a high degree of physiological plasticity and can acclimate to a variety of environmental conditions, but the transcriptomic response to acute UVR did not support this. In fact, *S. caninervis* had nearly twice as many DATs than *S. ruralis*, suggesting a more responsive and less constitutive response to UVR in the former. This result may be an indication that UVR radiation mediates other functions in *S. caninervis*, perhaps related to its habitat. In its natural desert habitat, this species experiences prolonged periods of desiccated quiescence seasonally, where it dissipates excess solar radiation as heat (Ekwealor et al., 2021). Since UV-B radiation varies substantially seasonally, relative to other regions of the spectrum (Robson et al., 2019), UV-B can act as a phenological cue for plants. For example, in *Populus tremula* interannual variation in UV-B radiation effects phenology and growth via UVR8-mediated ABA signaling with Flowering locus T (FT) genes (Strømme et al., 2015). The larger transcriptomic response in *S. caninervis*, along with the several differentially abundant uncharacterized protein transcripts, may suggest a phenological or other regulatory role of UVR in this species. Furthermore, the presence of three differentially abundant ELIPs in *S. caninervis* (but not *S. ruralis*) suggest a relationship between UVR and desiccation. It is possible that UVR is a phenological cue for seasonal desiccation in desert *S. caninervis*, though more research is needed to test this hypothesis. It will be interesting in the future to examine more genotypes in these species and their relatives from different habitat types, in a phylogenetic context, to explore how the expression patterns evolve.

## Data Availability Statement

The datasets presented in this study can be found in online repositories. The names of the repository/repositories and accession number(s) are as follows: https://github.com/jenna-tb-ekwealor/UV_syntrichia_acute and https://www.ncbi.nlm.nih.gov/ (BioProject PRJNA761650).

## Author Contributions

JE and BM conceptualized and designed the study. JE performed the research and wrote the initial draft. Both authors contributed to writing and editing.

## Conflict of Interest

The authors declare that the research was conducted in the absence of any commercial or financial relationships that could be construed as a potential conflict of interest.

## Publisher’s Note

All claims expressed in this article are solely those of the authors and do not necessarily represent those of their affiliated organizations, or those of the publisher, the editors and the reviewers. Any product that may be evaluated in this article, or claim that may be made by its manufacturer, is not guaranteed or endorsed by the publisher.

## Footnotes

1
www.polymerplastics.com/transparents_uvta_sheet.shtml

2
https://github.com/jenna-tb-ekwealor/UV_syntrichia_acute
