# Supplementary material for: Transcriptomic Effects of Acute Ultraviolet Radiation Exposure on Two Syntrichia Mosses
Source: Front Plant Sci. 2021 Oct 28;12:752913. doi: 10.3389/fpls.2021.752913 (PMC8581813; doi:10.3389/fpls.2021.752913)
Supplement: Supplementary file 1 [file Data_Sheet_1.docx]

Supplementary Material

**Table S1.** Per-sample read mapping rate in *Syntrichia ruralis* and *S. caninervis*.

**Table S2.** Significantly differentially abundant transcripts (absolute value log2-fold change of at least 1 and Benjamini-Hochberg adjusted *P*-value cut-off of 0.05) in *Syntrichia ruralis* with 10 minutes of UVR exposure.

**Table S3.** Significantly differentially abundant transcripts (absolute value log2-fold change of at least 1 and Benjamini-Hochberg adjusted *P*-value cut-off of 0.05) in *Syntrichia ruralis* with 30 minutes of UVR exposure.

**Table S4.** Significantly differentially abundant transcripts (absolute value log2-fold change of at least 1 and Benjamini-Hochberg adjusted *P*-value cut-off of 0.05) in *Syntrichia caninervis* with 10 minutes of UVR exposure.

**Table S5.** Significantly differentially abundant transcripts (absolute value log2-fold change of at least 1 and Benjamini-Hochberg adjusted *P*-value cut-off of 0.05) in *Syntrichia caninervis* with 30 minutes of UVR exposure.

**Table S6.** Significantly differentially abundant transcripts (absolute value log2-fold change of at least 1 and Benjamini-Hochberg adjusted *P*-value cut-off of 0.05) in both *Syntrichia ruralis* and *S. caninervis* after 30 minutes of UVR exposure.

**Table S7.** Transcripts that have a significantly different (Benjamini-Hochberg adjusted *P*-value cut-off of 0.05) abundance pattern in *Syntrichia ruralis* and *S. caninervis* after 10 and 30 minutes of UVR exposure, after controlling for differences at time 0.

**Table S8.** Clusters of transcripts that have similar abundance patterns over the timelines and have a significantly different (Benjamini-Hochberg adjusted *P*-value cut-off of 0.05) abundance pattern in *Syntrichia ruralis* and *S. caninervis* after 10 and 30 minutes of UVR exposure, after controlling for differences at time 0.

**Figure S1.** For each group of candidate gene families, ELIPs and LEAs, the simulated distribution of effect sizes from a Mann-Whitney *U* test comparing the absolute values of log2-fold changes of randomly selected groups of n transcripts to the full pool of transcripts in *Syntrichia ruralis* after 30 minutes of UVR exposure. Blue bars represent 95% of the distribution while grey bars fall outside of the 95% confidence interval. Vertical red lines indicate the actual effect size observed for the group of transcripts in question. Red lines that fall to the right of the 95% confidence interval are more differentially abundant than the null expectation, red lines within the interval are non-significant, and red lines to the left of the interval are less differentially abundant than the null expectation.

**Figure S2.** For each group of candidate gene families, ELIPs and LEAs, the simulated distribution of expected numbers of differentially abundant transcripts in *Syntrichia ruralis* after 30 minutes of UVR exposure. Blue bars represent 95% of the distribution while grey bars fall outside of the 95% credible set. Red asterisks indicate the actual number of differentially abundant transcripts in that group of transcripts. Red asterisks that fall within the 95% credible set are non-significant while those that fall outside of the 95% credible set indicate groups that have significantly more differentially abundant transcripts than expected by chance.

**
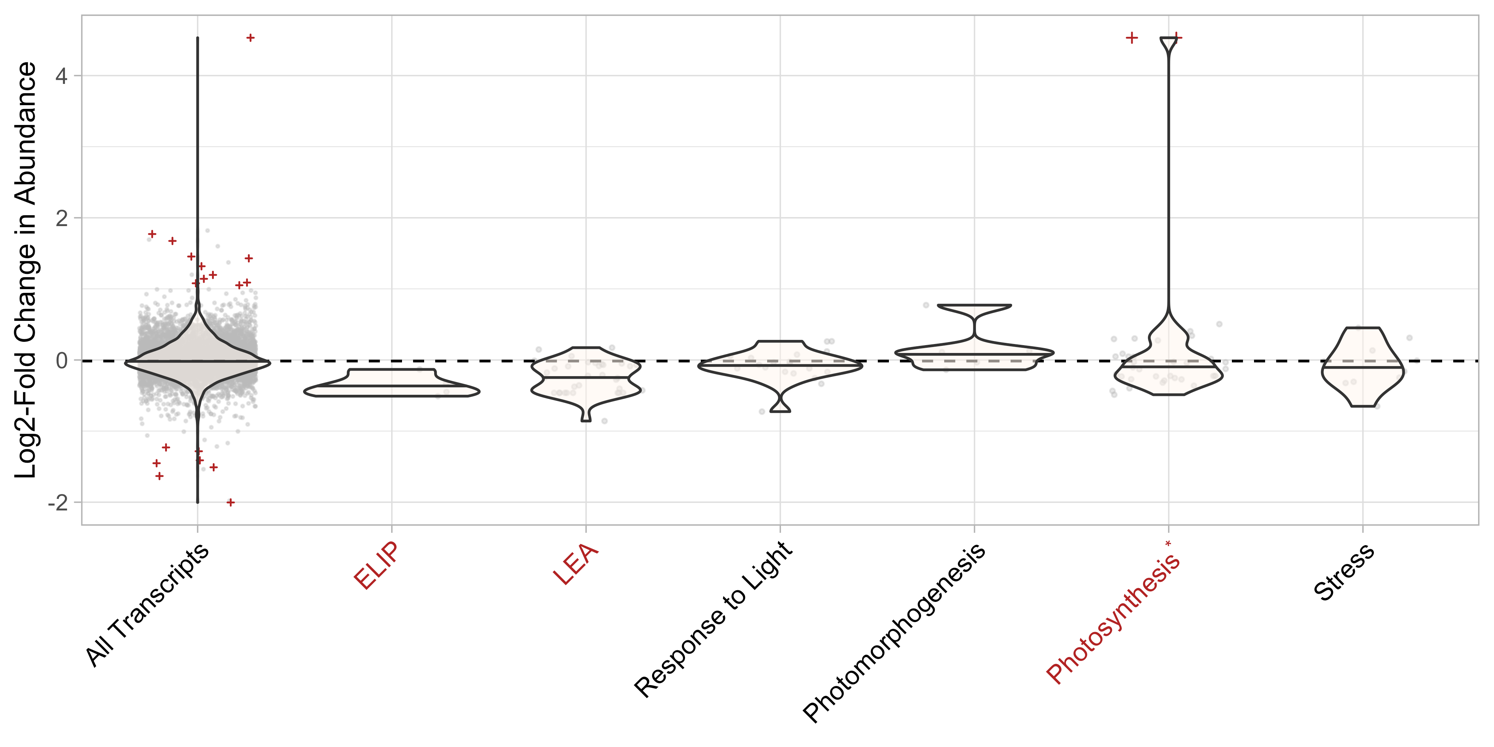
**

**Figure S3.** Differential transcript abundance of candidate gene groups after 10 minutes of UV radiation exposure in *Syntrichia ruralis.* Positive values correspond to increased transcript abundance after 30 minutes of UV radiation treatment, negative values correspond to decreased transcript abundance. Violin plots represent the log2-fold change (LFC) value for the gene groups and grey points correspond to LFC of individual transcripts. Transcripts that are significantly differentially abundant (*P*-adj < 0.05, absolute-value LFC > 1) are labeled with red crosses and groups with absolute-value LFC distributions that are significantly larger than that of the whole transcriptome (All Transcripts) are labeled in red on the x-axis. Groups with significantly more differentially abundant transcripts than expected by change are labeled with an asterisk on the x-axis.

**Figure S4.** For each group of candidate gene families, ELIPs and LEAs, and transcripts with the GO terms *photosynthesis* and *light,* the simulated distribution of effect sizes from a Mann-Whitney *U* test comparing the absolute values of log2-fold changes of randomly selected groups of n transcripts to the full pool of transcripts in *Syntrichia ruralis* after 10 minutes of UVR exposure. Blue bars represent 95% of the distribution while grey bars fall outside of the 95% confidence interval. Vertical red lines indicate the actual effect size observed for the group of genes in question. Red lines that fall to the right of the 95% confidence interval are more differentially abundant than the null expectation, red lines within the interval are non-significant, and red lines to the left of the interval are less differentially abundant than the null expectation.

**Figure S5.** For each group of candidate gene families, ELIPs and LEAs, and transcripts with the GO terms *­photosynthesis* and *stress*, the simulated distribution of expected numbers of differentially abundant transcripts in *Syntrichia ruralis* after 10 minutes of UVR exposure. Blue bars represent 95% of the distribution while grey bars fall outside of the 95% credible set. Red asterisks indicate the actual number of differentially abundant transcripts in that group of transcripts. Red asterisks that fall within the 95% credible set are non-significant while those that fall outside of the 95% credible set indicate groups that have significantly more differentially abundant transcripts than expected by chance.

**Figure S6.** For each group of candidate gene families, ELIPs and LEAs, the simulated distribution of effect sizes from a Mann-Whitney *U* test comparing the absolute values of log2-fold changes of randomly selected groups of n transcripts to the full pool of transcripts in *Syntrichia caninervis* after 30 minutes of UVR exposure. Blue bars represent 95% of the distribution while grey bars fall outside of the 95% confidence interval. Vertical red lines indicate the actual effect size observed for the group of genes in question. Red lines that fall to the right of the 95% confidence interval are more differentially abundant than the null expectation, red lines within the interval are non-significant, and red lines to the left of the interval are less differentially abundant than the null expectation.

**Figure S7.** For each group of candidate gene families, ELIPs and LEAs, the simulated distribution of expected numbers of differentially abundant transcripts in *Syntrichia ruralis* after 10 minutes of UVR exposure. Blue bars represent 95% of the distribution while grey bars fall outside of the 95% credible set. Red asterisks indicate the actual number of differentially abundant transcripts in that group of transcripts. Red asterisks that fall within the 95% credible set are non-significant while those that fall outside of the 95% credible set indicate groups that have significantly more differentially abundant transcripts than expected by chance.

**
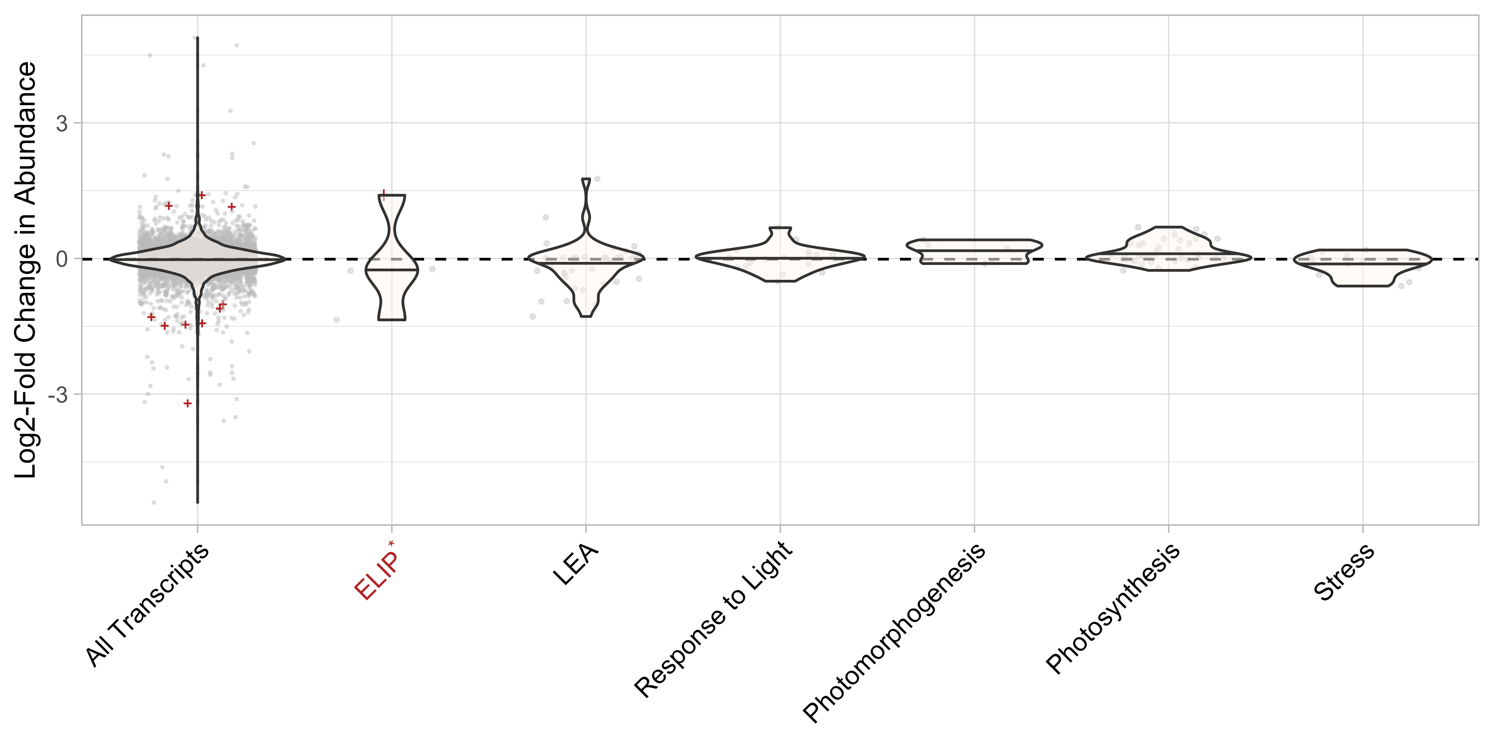
**

**Figure S8.** Differential transcript abundance of candidate gene groups after 10 minutes of UV radiation exposure in *Syntrichia caninervis.* Positive values correspond to increased transcript abundance after 10 minutes of UV radiation treatment, negative values correspond to decreased transcript abundance. Violin plots represent the log2-fold change (LFC) value for the gene groups and grey points correspond to LFC of individual transcripts. Transcripts that are significantly differentially abundant (*P*-adj < 0.05, absolute-value LFC > 1) are labeled with red crosses and groups with absolute-value LFC distributions that are significantly larger than that of the whole transcriptome (All Transcripts) are labeled in red on the x-axis. Groups with significantly more differentially abundant transcripts than expected by change are labeled with an asterisk on the x-axis.

**Figure S9.** For each group of candidate gene families, ELIPs and LEAs, the simulated distribution of effect sizes from a Mann-Whitney *U* test comparing the absolute values of log2-fold changes of randomly selected groups of n transcripts to the full pool of transcripts in *Syntrichia caninervis* after 10 minutes of UVR exposure. Blue bars represent 95% of the distribution while grey bars fall outside of the 95% confidence interval. Vertical red lines indicate the actual effect size observed for the group of genes in question. Red lines that fall to the right of the 95% confidence interval are more differentially abundant than the null expectation, red lines within the interval are non-significant, and red lines to the left of the interval are less differentially abundant than the null expectation.

**Figure S10.** For each group of candidate gene families, ELIPs and LEAs, the simulated distribution of expected numbers of differentially abundant transcripts in *Syntrichia caninervis* after 10 minutes of UVR exposure. Blue bars represent 95% of the distribution while grey bars fall outside of the 95% credible set. Red asterisks indicate the actual number of differentially abundant transcripts in that group of transcripts. Red asterisks that fall within the 95% credible set are non-significant while those that fall outside of the 95% credible set indicate groups that have significantly more differentially abundant transcripts than expected by chance.
